# Association of Histologic Growth Patterns and Some Histopathologic Features in Clear Cell Renal Cell Carcinoma with Patient’s Survival after Nephrectomy: A Cross-Sectional Study

**DOI:** 10.30699/ijp.2025.2033228.3319

**Published:** 2025-03-10

**Authors:** Mehdi Farzadnia, Naser Tayyebi Meibodi, Ahmad Jafari Joshaghan, Mahnaz Baradaran, Motahare Ebrahimnejad, Saeid Dehghan Nezhad, Farideh Ranjbar

**Affiliations:** 1 *Cancer Molecular Pathology Research Center* *٫* * Mashhad University of Medical Sciences* *٫* * Mashhad* *٫* * Iran *; 2 *Cutaneous Leishmaniasis Research Center, Mashhad University of Medical Sciences, Mashhad, Iran *; 3 *Department of pathology, Faculty of Medicine, Mashhad University of Medical Sciences, Mashhad, Iran. *; 4 *Department of Pathology, School of Medicine, Loghman Hakim Hospital, Shahid Beheshti University of Medical Sciences, Tehran, Iran*; 5 *Department of Pathology, Faculty of Medicine, Mashhad University of Medical Sciences, Mashhad, Iran*

**Keywords:** Carcinoma, Clear Cell Renal Cell Carcinoma, Histologic Growth Patterns, Survival, Nephrectomy

## Abstract

**Background & Objective:**

Clear cell renal cell carcinoma (CCRCC) is the most common type of renal cancer. Limited studies have been conducted about factors affecting the survival of patients with CCRCC. In this study, we aimed to evaluate the association between histologic growth patterns (HGPs) and some pathologic features and survival in CCRCC.

**Methods:**

This cross-sectional study evaluated the association between HGPs and other pathologic features and the survival of 145 patients with CCRCC after nephrectomy in Emam-Reza Hospital (Mashhad, Iran) from 2012 to 2022. Two expert pathologists assessed HGPs and other pathologic features, like cytopathologic changes. All analyses were performed using IBM SPSS version 26 software. A *P* value less than 0.05 was considered statistically significant.

**Results:**

In the current study, we assessed the association of the 6 most prevalent growth patterns with the patient’s survival. Some clinicopathologic features like tumor stage and grade, tumor size, and necrosis are negatively linked with survival. Two important cytologic features, including sarcomatoid and rhabdoid, were also associated with survival time in patients with CCRCC (P values < 0.05). Regardless of the nuclear grade of the tumor, some patterns like solid sheet, papillary, and thick trabecular were associated with lower survival.

**Conclusion:**

Some HGPs are significantly associated with the patient’s survival in CCRCC. A greater variety of patterns within each specimen has been associated with a reduced survival rate. The impact of HGPs on patient survival may be as significant as the nuclear grade.

## Introduction

Clear cell renal cell carcinoma (CCRCC) is the most common type of renal cell carcinoma (RCC). The origin of clear cell carcinoma is proximal tubular cells, and the incidence of metastasis in this type is approximately 15% (1-3). Among other types of RCC, CCRCC has the worst prognosis (4, 5). Recurrence occurs in more than one-third of cases of localized CCRCC after surgical treatment, and local or distant metastasis subsequently appears in other cases (6, 7). The most common genetic change in CCRCC is the loss of the short arm of chromosome 3, encoding the *VHL* gene. Gain of 14q, 4p, and 9p indicates a poor prognosis, whereas gain of 5q31 has been associated with long-term survival. Mutation of the *PBRM1*, *BAP1*, and *STED2* genes also plays a role in the development of morphological diversity, more aggressive phenotypes, and poor prognosis in CCRCC (8, 10). Histopathological analysis is the criterion standard for diagnosis and prognosis determination, but the wide histological diversity of this tumor is challenging (8, 9). To date, the morphological complexity has been limited to tumor stage, grade, and necrosis. However, these factors do not encompass all behavioral aspects of the disease, and how other phenotypes affect patients' prognosis is poorly understood. Clear cell renal carcinoma was classified into 4 grades and stages (1 to 4) based on the classification of the World Health Organization/ International Society of Urological Pathology (WHO/ISUP) 2016 grading system and the American Joint Committee on Cancer (AJCC) TNM staging, respectively (26). The WHO/ISUP described 4 descriptive grades (1 to 4) based on increased nucleus size, nuclear irregularity, and nucleolus prominence (10-13). Grade 4 is defined by cytological features such as sarcomatoid, rhabdoid, giant syncytial tumoral cell, or nucleus pleomorphism (14-17).

The role of some histological growth patterns (HGPs) in the prognosis of CCRCC has been determined (14, 18, 19). Tumor necrosis is an independent predictive factor for overall survival and metastasis-free survival in CCRCC patients (20-22). Evaluation of histopathological diversity can clarify tumor behavior and provide additional prognostic models (23). Although studies have shown the effects of some histopathological features in CCRCC, further research is needed to assess the impact of different HGPs on survival, particularly in comparison with known prognostic factors (3, 24, 25). In this study, we examined the relationship between HGPs of CCRCC and patient survival after nephrectomy.

## Materials and Methods

This retrospective cross-sectional study included 145 patients who were referred to Emam-Reza Hospital in Mashhad, Iran, from 2012 to 2022 and pathologically diagnosed with CCRCC after nephrectomy.

Histological growth patterns and cytological features were evaluated for each sample, regardless of previous reports. The 6 common HGPs in this study were:

- Compact small nests (cell nests containing 1 to 2 rows of epithelial cells with distinct boundaries, separated by delicate fibrovascular septa)
- Tubular/acinar (elongated gland-like structures covered with 1 to 2 rows of epithelial cells placed around a lumen-like space)
- Thick trabecular (large elongated cell nests containing 3 or more rows of epithelial cells, separated by discontinuous fibrovascular septa)
- Solid sheets (large cellular plates without distinct fibrovascular septa)
- Papillary (finger-like structures with a distinct fibrovascular core covered by cuboid or columnar epithelial cells)
- Cystic (large dilated lumens often containing red blood cells or eosinophilic secretions, covered by a single row of flattened epithelial cells)

Figures 2 and 3 show all the HGPs and cytologic features of CCRCC in our study.

**Fig.1:**
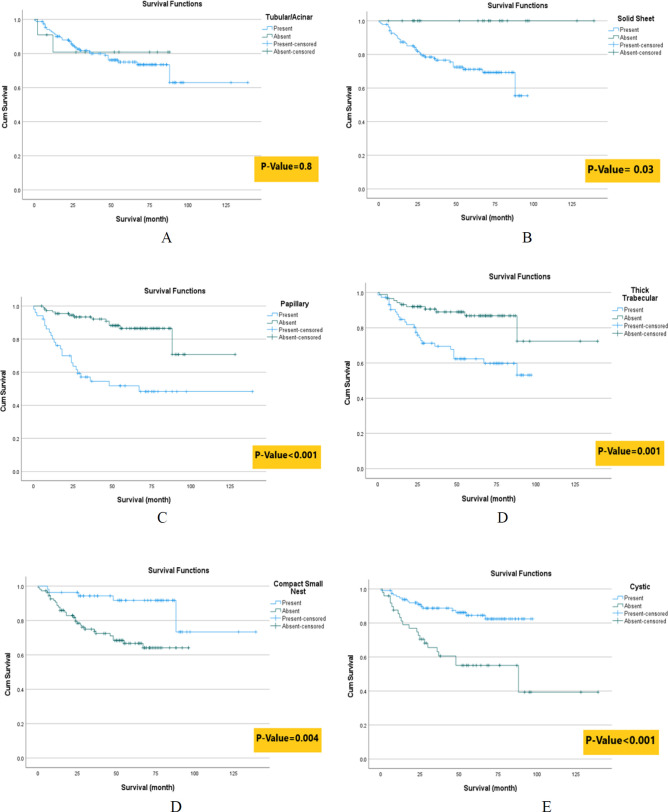
Kaplan Meier survival analyses according to different HGPs include A) Tubular/Acinar, B) Solid Sheet, C) Papillary, D) Thick Trabecular, E) Compact Small Nest, F) Cystic patterns

Patients’ pathological specimens with a clinical diagnosis of CCRCC were assessed based on standard protocols and examined with standard hematoxylin and eosin staining. Two expert pathologists independently reviewed the slides, and in the presence of discrepancies, they reviewed the slides together, and a consensus was reached. Diagnostic immunohistochemistry (IHC) panels were used in doubtful cases to confirm the diagnosis of CCRCC and rule out other renal epithelial cell neoplasms with clear cell features, such as clear cell papillary RCC, and some chromophobe, papillary RCC, or even oncocytoma. (Figure 1 shows one of the samples with mixed papillary and clear cell features that confirmed the diagnosis of CCRCC according to IHC findings.)

The exclusion criteria were cases of RCC with a clear cell appearance that were not included in the CCRCC group according to the immunohistochemical panel (including CK7, CD10, CD117, and AMACR) performed for suspicious cases and mimic types. Cases with incomplete hospital-documented records and low-quality pathological slides or blocks indicating morphological changes were also excluded.

Survival time was calculated in months, from the time of nephrectomy until death due to cancer or any cancer-related cause and its complications, or until the time of statistical analysis for live patients. For this purpose, we used the documented records of patients in the hospital. Finally, the correlation between HGPs and clinicopathological features with patient survival was assessed.

Our ethical committee approved the proposal, which follows the ethical code of IR.MUMS.MEDICAL.REC.1402.076.

### Statistical analysis

Mean, standard deviation, median, and range were used to describe quantitative data, and frequency and percentage were used for qualitative variables. Appropriate tables and graphs were used to describe the data. Univariate and multiple Cox regression analyses were conducted to identify factors associated with patient survival. Survival diagrams were made using the Kaplan-Meier method to compare survival rates. All analyses were performed using IBM SPSS version 26 software. A *P* value less than 0.05 was considered statistically significant.

**Table 1:** The demographic, clinical, and pathological characteristics of patients

Characteristics	Levels	Frequency/ Mean ± SD	Percent %
Gender	Male	88	(60.7%)
	Female	57	(39.3%)
Age (year)		57.5 ± 12.17	
Death		37	(25.5%)
Survival Time After Surgery (month)		50.69 ± 27.93	
Nephrectomy	Radical	98	(67.6%)
	Partial	47	(32.4%)
Kidney Side	Right	91	(62.8%)
	Left	54	(37.2%)
Tumor Stage	Stage 1	74	(51.0%)
	Stage 2	20	(13.8%)
	Stage 3	47	(32.4%)
	Stage 4	4	(2.8%)
Fuhrman Nuclear Grade	Grade 1	24	(16.6%)
	Grade 2	70	(48.3%)
	Grade 3	28	(19.3%)
	Grade 4	23	(15.9%)
Tumor Size (cm)		6.25 ± 3.45	
Necrosis (%)		11.44 ± 14.95	
Histologic Growth Patterns (HGPs) number		5.67 ± 1.97	
Metastasis		17	(11.7%)
Recurrence		19	(15.4%)

## Results

In this study, all slides of 145 patients with CCRCC were reviewed. Demographic, clinical, and pathological characteristics of patients are shown in Table 1. The mean age of patients was 57.5 ± 12.17 years, and 88 patients (60.7%) were male. Thirty-seven (25.4%) patients died. Table 2 shows all of the HGPs. Cystic, tubular/acinar, solid sheet, papillary, thick trabecular, and compact small nest patterns were studied in our research as more common patterns in our samples, and the most prevalent pattern was the tubular/acinar pattern (89.7%).

In the univariate Cox regression analysis (Table 3), several factors were significantly associated with patient survival. Increasing age was associated with poorer survival (HR: 1.033; 95% CI, 1.006–1.060; *P* = 0.016). Tumor size also showed a significant positive association with the hazard of death (HR: 1.001; 95% CI: 1.000–1.002; *P* < 0.001). Lower nuclear grades (grades 1/2 and 3) were associated with a significantly reduced risk of death compared with grade 4 (HR: 0.069; 95% CI: 0.031–0.156; *P* < 0.001, and HR: 0.302; 95% CI: 0.139–0.655; *P* = 0.002, respectively). Similarly, lower tumor stages (stages 1 and 2) were strongly associated with improved survival compared with stage 4 (HR: 0.021; 95% CI: 0.004–0.105; *P* < 0.001, and HR: 0.139; 95% CI: 0.032–0.600; *P* = 0.008, respectively). Tumoral necrosis was also a significant risk factor, with a hazard ratio of 0.031 (95% CI: 0.004–0.229; *P* = 0.001). Gender and kidney laterality did not demonstrate statistically significant associations with survival in the univariate analysis.

The survival rate of patients with solid sheet, papillary, and thick trabecular patterns was significantly lower than that of patients without these patterns. However, the survival rate of patients with compact small nests and cystic patterns was greater than that of patients without these patterns. There was no significant difference in survival rate between patients with and without tubular patterns. Diagram 1 and Table 3 show the survival analyses according to different HGPs.

Patients with sarcomatoid and rhabdoid cytologic features had a lower survival rate than those without these (diagrams 2A-B and Table 1). We found a negative association of Fuhrman's nuclear grade, primary tumor stage (pT stage), and necrosis with patients' survival time. As the nuclear grade and tumor stage increased, the survival time of patients decreased (diagrams 2C-E). Diagram 2F shows a significant difference between the survival rate of patients with more variable morphologies. Survival tends to decrease as the number and variety of tissue types increases.

In the multiple Cox regression analysis, adjusted for potential confounders (Table 4), tumor stage remained a strong predictor of survival. Patients with stage 1 disease had a significantly reduced risk of death compared with those with stage 4 (HR: 0.033; 95% CI: 0.004–0.277; *P* = 0.002). Similarly, patients with grade 1 or 2 tumors exhibited a markedly lower risk of death compared with those with grade 4 tumors (HR: 0.066; 95% CI: 0.005–0.819; *P* = 0.034). Among the histologic growth patterns, the presence of a cystic pattern was associated with better survival (HR: 1.345; 95% CI: 0.312–2.541; *P* = 0.031), and the presence of a solid sheet pattern was associated with poorer survival (HR: 0.406; 95% CI: 0.196–0.841; *P* = 0.015). However, other growth patterns, such as compact small nests, papillary, and thick trabecular, did not reach statistical significance in the adjusted model. The presence of sarcomatoid and rhabdoid cytologic features was also associated with poorer survival. Age, gender, tumor size, and kidney side were not significant predictors of survival in the multiple analyses.

To better illustrate the importance of HGPs in patient survival, we presented the combined HGPs/grade model. For this purpose, nuclear grades 1 and 2 are considered low grades, whereas nuclear grades 3 and 4 are regarded as high grades. The HGPs were divided into 3 categories according to the dominant pattern in each sample. HGPs associated with higher survival rates (including cystic and compact small nests) were considered 1) less aggressive patterns, HGPs associated with lower survival rates (including thick trabecular, solid sheet, and papillary) were considered 2) more aggressive patterns, and the tubular/acinar pattern, which was not significantly related to patient survival, was considered 3) intermediate pattern. In this way, each of the 3 categories of patterns was examined with each category of nuclear grade (high/low). The results of diagram 3 indicate that more aggressive patterns (including thick trabecular, solid sheet, and papillary patterns) exhibited the lowest survival rates at any nuclear grade compared with less aggressive and intermediate patterns, even with high nuclear grade.

**Table 2:** Different HGPs and morphologies of CCRCC

HGPs and cytopathologic features	Frequency	Percent	Survival Time After surgery	
			Mean	SD
**Cystic**	102	(70.3%)	52.21	25.19
**Tubular/acinar**	130	(89.7%)	51.06	27.93
**Alveolar**	45	(31.0%)	42.95	33.1
**Solid Sheet**	122	(84.1%)	40.83	25.83
**Papillary**	49	(33.8%)	47.27	32.3
**Compact Small Nests**	50	(34.5%)	61.46	29.84
**Large Nests**	45	(31.0%)	55.81	30.05
**Thick Trabecular**	65	(44.8%)	48.5	28.6
**Bleeding Follicles**	43	(29.7%)	55.14	25.17
**Sarcomatoid**	8	(5.5%)	34.5	23.46
**Rhabdoid**	20	(13.8%)	27	22.43

**Table 3:** Result of univariate Cox regression analysis of predictors for patients survival.

		B	Crude HR	95.0% CI for HR		P-value
				Lower	Upper	
Gender^1^	Female	0.159	1.172	0.620	2.217	0.625
Age		0.032	1.033	1.006	1.060	0.016
Kidney Side^2^	Right	-0.136	0.873	0.458	1.665	0.680
Tumor Size (cm)		0.001	1.001	1.000	1.002	<0.001
Nuclear Grade^3^						
	Grade 1/2	-2.669	0.069	0.031	0.156	<0.001
	Grade 3	-1.197	0.302	0.139	0.655	0.002
Tumor Stage^4^						
	Stage 1	-3.884	0.021	0.004	0.105	<0.001
	Stage 2	-1.976	0.139	0.032	0.600	0.008
	Stage 3	-0.998	0.369	0.110	1.240	0.107
Necrosis^5^		-3.463	0.031	0.004	0.229	0.001
Compact Small Nest^5^		-1.282	0.278	0.115	0.669	0.004
Tubular/Acinar^5^		0.191	1.211	0.291	5.029	0.801
Thick Trabecular/Insular^5^		1.163	3.201	1.585	6.461	0.001
Papillary^5^		1.547	4.696	2.426	9.090	<0.001
Cystic^5^		-1.263	0.283	0.148	0.540	<0.001
Solid Sheets^5^		3.327	27.862	0.768	1010	0.039
Sarcomatoid^5^		1.207	3.343	1.295	8.635	0.013
Rhabdoid^5^		2.070	7.922	4.028	15.581	<0.001

**B**: Regression Coefficient. **HR**: Hazard Ratio. **CI**: Confidence Interval. **Reference Levels: 1: **Male**, 2: **Left**, 3: **Grade 4**, 4: **Stage 4, **5: **Absent**.**

**Table 4:** Results of multiple Cox regression analysis adjusted for the possible confounders.

		B	Adjusted HR	95.0% CI for HR		P-value
				Lower	Upper	
Gender^1^	Female	0.035	1.035	0.483	2.219	0.929
Age		0.021	1.021	0.993	1.050	0.145
Kidney Side^2^	Right	-0.249	0.779	0.361	1.684	0.526
Tumor Size (cm)		0.000	1.000	0.999	1.001	0.703
Compact Small Nest^3^	Present	-0.934	0.393	0.133	1.160	0.091
Tubular/Acinar^3^	Present	-0.236	0.790	0.095	6.602	0.828
Thick Trabecular/Insular^3^	Present	0.137	1.147	0.492	2.673	0.751
Papillary^3^	Present	0.219	1.245	0.468	3.317	0.661
Cystic^3^	Present	-0.901	0.406	0.196	0.841	0.015
Solid Sheet^3^	Present	0.876	1.345	0.312	2.541	0.031
Sarcomatoid^3^	Present	1.649	0.692	0.029	1.273	0.037
Rhabdoid^3^	Present	1.283	0.577	0.028	2.778	0.041
Nuclear Grade^4^						
	Grade 1/2	-2.720	0.066	0.005	0.819	0.034
	Grade 3	-2.058	0.128	0.011	1.550	0.106
Tumor Stage^5^						
	Stage 1	-3.408	0.033	0.004	0.277	0.002
	Stage 2	-1.891	0.151	0.022	1.040	0.055
	Stage 3	-1.295	0.274	0.052	1.430	0.125

**B**: Regression Coefficient.

**HR**: Hazard Ratio.**CI**: Confidence Interval.

**Reference Levels: 1: **Male**, 2: **Left**, 3: **Absent**, 4: **Grade 4**, 5: **Stage 4**.**

**Fig. 2:**
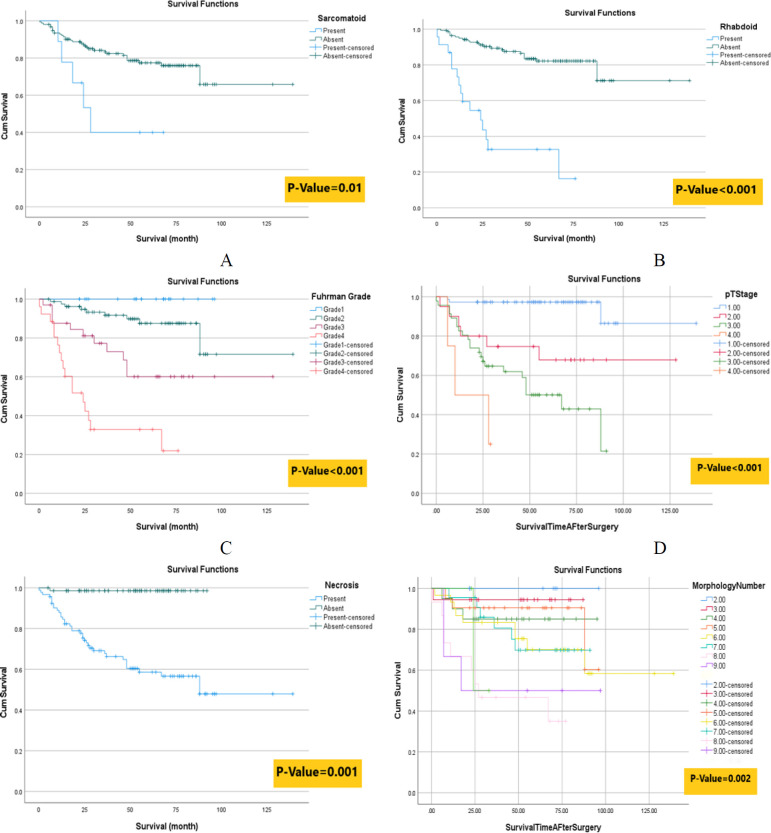
Kaplan Meier survival analyses according to A) Sarcomatoid, B) Rhabdoid cytologic features C) Nuclear Grade, D) Primary Tumor Stage (PT Stage), E) Necrosis, and F) HGP number. The definitions of each color are seen on the right side of the diagrams. For example, in diagram 2F, if a specimen had 2 different patterns (first blue line), the patient had a higher survival rate than a patient with nine different patterns (purple line).

**Fig. 3:**
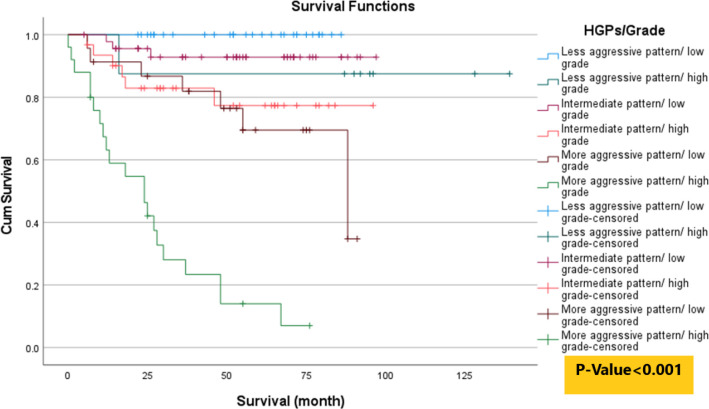
Kaplan Meier survival (based on log-rank test) analyses according HGPs/Grade model

## Discussion

lCCRCC has a higher mortality rate than chromophobe and papillary RCC. Histopathological analysis is a standard method used for diagnosing and determining the prognosis of RCC.

The present study evaluated the clinicopathologic features and HGPs of CCRCC with patient survival. A total of 145 patients were assessed. Compact small nests and cystic (less aggressive) patterns are associated with higher survival rates. In contrast, papillary, solid sheet, and thick trabecular (more aggressive) patterns and diversity of patterns are significantly correlated with lower survival rates. Each nuclear grade (high or low) of more aggressive patterns had a lower survival rate than less aggressive patterns, even with a high grade.

Previous studies have indicated that patients with higher tumor stages and grades experience shorter survival rates (5, 27, 28). Our study has shown similar results, but we also showed the impact of HGPs compared with nuclear grade on patient survival. However, by examining the effect of confounding factors in multiple Cox regression analysis, only the cystic pattern was favorably correlated with patient survival, and the solid pattern was unfavorably correlated with survival.

Sirohi et al. found that expansile nests, small nests, and nests with a high nuclear-to-cytoplasmic ratio were associated with good outcomes, and solid sheets, spindled low-grade patterns, sarcomatoid, and rhabdoid features were associated with poor outcomes (14). In the current study, similar to Sirohi et al., the solid sheet, sarcomatoid, and rhabdoid patterns were associated with a lower survival rate.

Andreiana et al. mentioned that sarcomatoid transformation was the most common histopathologic change in a higher stage of CCRCC, but it was not associated with the disease stage. They found that the disease stage was associated with Fuhrman’s nuclear grade. Most of the patients had a mixed pattern, which was significantly related to the advanced stage of the disease (29). We showed that some HGPs were associated with patients’ survival and higher nuclear grade and tumor stage were associated with poorer survival. The causes of the difference between our results and Andreiana et al.'s study in terms of examining the association between sarcomatoid transformation and stage could be that their sample size was smaller than our study; they examined 75 cases, whereas we had 145 cases.

Verine et al. demonstrated that cystic, compact, large nest, acinar, papillary, and alveolar patterns had higher survival rates than rhabdoid and sarcomatoid patterns in CCRCC (30). We demonstrated that any nuclear grades of papillary, trabecular, and solid sheet patterns had lower survival rates than cystic and small nest patterns.

Yang et al. concluded that micropapillary, solid, and hobnail patterns were associated with poorer outcomes in papillary RCC (31). However, we did not assess patients with papillary RCC, but the results of both studies emphasize the important role of histopathologic patterns of RCC on survival. This issue should be investigated in future studies.

This was a cross-sectional study, and all the slides and blocks that were available from CCRCC patients over the last 10 years were reviewed. Our study had some limitations, such as incomplete hospital records for patients' past medical and treatment history, and not considering other prognostic pathologic factors such as venous and lymph node invasion. Nevertheless, demonstrating the importance of HGPs in patient survival compared with other proven prognostic factors, such as nuclear grade, was a strong point. So far, studies on this topic have been limited, and further research is needed.

## Conclusion

It is concluded that assessing HGPs in CCRCC is an important prognostic factor for patient survival. In addition, the subtype of HGPs, regardless of the nuclear grade, can be important in patient outcomes. 

## Data Availability

Data are available upon reasonable request from the corresponding author.
